# Dr. Burne on Stricture of the Sigmoid Flexure of the Colon

**Published:** 1830-07-01

**Authors:** 


XXVIII.

### STRICTURE OF THE SIGMOID FLEXURE OF THE COLON.*

Dr. Burne, of this metropolis, has published two interesting cases of contraction of the colon in our esteemed provincial contemporary. This class of affections is not sufficiently rare to be a matter of curiosity, but common enough to be one of practical interest.

*Case* 1. A gentleman, between 45 and 50 years of age, of sanguine temperament and a free liver, consulted Dr. Burne in February, 1826, on account of troublesome superficial ulcerations in the mouth, heat and dryness in the mouth and pharynx, and some little trouble in deglutition. He was feverish, but the bowels were habitually regular. Our author intimated to his patient a fear of incipient stricture of the œsophagus, and prescribed leeches and saline aperients, under the use of which the ulcers soon healed. In November, and again in February, 1827, the patient applied to Dr. B. for dyspeptic symptoms, after which he saw no more of him until February, 1828, when he still suffered from the dyspepsia, with the addition of a sluggish state of bowels. In July, 1828, he returned with an aggravation of the complaint, and was troubled much with fulness when the bowels were not freely moved, which was now effected with more difficulty. The Cheltenham waters were tried, with great but temporary benefit, for after he left the place, the irregularity of the bowels and dyspepsia were re-established, and his age and sallow face excited an apprehension of organic disease. A consultation was proposed and Dr. Armstrong was called in, but nothing conclusive was elicited. The costiveness continuing, Dr. Burne determined to ascertain by manual examination, if any cause of obstruction existed in the rectum. On passing the finger forward as far as was practicable, he met with a hard immoveable tumour, the size of an egg, and still further backwards and upwards the tip of the finger reached a contraction of the bowel, not larger in diameter than a swan quill, surrounded by a hard knotty structure, which altogether resembled a scirrhous os uteri. The opinion of the late Mr. Wadd was now taken on the propriety of using bougies; he decided against them. Severe spasmodic contractions of the bowels suc-

* Midland Reporter, No. VIII. May, 1830.

ceeded the exhibition of castor oil, and these again were followed by a sharp attack of inflammation, through which the patient with difficulty struggled. Mr. Copeland, Mr. Brodie, and Sir Astley Cooper were consulted, but all ultimately agreed on the hopelessness of the case, and the inexpediency of surgical interference. The patient lingered on, scarcely ever passing any feculent matter, and suffering from occasional attacks of violent spasm and succeeding inflammation till the 20th of February, 1829, on which day the spasms were followed by sudden pain in the left side of the belly, compared to the discharge of a pistol. The abdomen immediately became tense, the powers sank, and in eleven hours the unfortunate patient expired.

*Sectio Cadaveris.* "The abdomen being opened, a large quantity of feculent matter, of soft consistence, was seen lying among the intestines and upon the mesentery, and was found to proceed from a transverse rupture of the colon, about an inch long, at the spot from whence the violent pain darted. The whole of the colon was filled with feculent matter of the same kind; the sigmoïd flexure was seen stretching across the brim of the pelvis to the right side, when it turned quickly upon itself and terminated in the diseased portion, which was situated directly under the promontory of the sacrum. The diseased part was about the size of an egg, and consisted of a scirrhous degeneration of those strictures of the intestine situated between the mucous and peritoneal coats. The aperture of communication between the colon and the rectum through the diseased part, scarcely equalled the size of a swan quill, and had a curved direction, which proved the correctness of the opinion, that force used in attempts to pass a bougie would be likely to rupture the bowel. The lower opening looked backwards and downwards to the hollow of the sacrum, and its margin was knotted and irregular as has been described. There were adhesions of the sigmoid flexure to the small intestines, and the scirrhous mass was adherent to the sacrum."

*Case* 2. A female pauper in Covent Garden Workhouse had been admitted, three weeks before her death, in a state of extreme emaciation and with a remarkably distended abdomen; she passed scarcely any feculent matter during the three weeks and was constantly vomiting, so that nothing except brandy and water could be retained upon the stomach. This is all the account of the case that Dr. Burne could procure.

*Sectio Cadaveris.* "Before the abdomen was opened, traces of the convolutions of the intestines were evident, by corresponding elevations of the integuments: these convolutions were found to be distended with gas, and the colon was full throughout of soft feculent matter; and at the termination of the sigmoid flexure in the rectum, was a circular contraction of the bowel forming the annular stricture. There was no thickening or disease about the part, and the contraction had the appearance of the bowel tied with a ligature, except that there were neither folds nor puckering."

Many interesting observations on the foregoing cases, and on stricture of the colon generally, are made by Dr. Burne. He particularly remarks the soft consistence of the feculent matter, a circumstance which does not appear to have been noticed by systematic writers, but which he believes to be generally present in cases of this disease. Our author looks on it as a special provision of Nature, but we must be excused if we doubt the correctness of his opinion, and hesitate to attribute such an office to the vis medicatrix naturæ. Dr. Burne appears to regret that, in the first case, no attempt was made to establish an artificial anus. We cannot share in his concern, but agree with the late Mr. Wadd, who opposed the proposition. Setting aside the dangers and difficulties of the operation, neither of them inconsiderable, where would have been the service of joining a most loathsome to an incurable disease, of superadding disgust to commiseration in the minds of the patient's family and friends? The following remarks on purgative medicines are judicious.

"All are agreed that the aperients which must be necessarily given, should be of a mild character, and the recommendations of authors who have treated on the subject, are limited to castor oil, senna, and sulphur; thus leaving unnoticed saline aperients, which, as will presently be seen, are the most efficacious. These medicines, castor oil, senna and sulphur, although desirable from their mild qualities, are very uncertain and ineffectual in cases of stricture: it is true they promote a moderate, and so far, a proper peristaltic action of the intestines, but as they do not render the fæces watery, this action is not followed by sufficient evacuation, and therefore not by sufficient relief. Sulphur is objectionable on other grounds; it has been known to form into balls when taken in large doses, and in this way may add to the mischief. The same objection applies also to magnesia, which has been found accumulated in a large quantity above the stricture.

"While the subject of the first case was at Cheltenham and taking the waters, the evacuations were so thin that the colon emptied itself effectually every day, and under these favourable circumstances the patient lost all complaint and improved surprisingly. This first suggested to me the use of saline aperients, which were given in the form of Seidlitz powders and of sulphate of magnesia, in a very diluted solution; and they were found to operate much more pleasantly and efficiently than other aperients. These, however, and the Cheltenham water itself drunk in town, were by no means so certain in their operation as the waters drunk at Cheltenham, owing, no doubt, to the want of auxiliary circumstances which are known to favour the operation of mineral waters, as change of scene, absence from the fatigue and anxiety of business, early rising, and exercise. On one occasion, when castor oil was substituted for salts, its effect was exceedingly injurious; it duly excited the action of the intestines, but as it did not render the fæces watery, they could not pass the stricture freely, and the consequence was violent spasmodic pain and vomiting.

"Drastic and heating purgatives are very properly objected to in all cases of stricture; nevertheless, the distress of the patient on one occasion was so great for the want of evacuations, that a person of very great practical attainments was induced to propose the administrations of croton oil, the propriety of which was much discussed, on account of its irritating properties and violent action; but its employment being much urged by the proposer on the score of its unrivalled purgative power in other cases, it was exhibited in the dose of one drop, which was repeated in the space of half an hour. The effect, as was anticipated, was nearly fatal; it produced most violent contractions of the intestines, and spasmodic pains, with a distressing heat along the whole alimentary canal, and constant and urgent, but ineffectual efforts to go to stool, the scanty evacuation consisting of nothing more than a bloody secretion from the rectum, the product of excessive irritation. The violent action of the intestines led one to fear a rupture of the colon, of which the sequel of the case proved there was great danger.

"In the medical treatment of stricture of the large intestine, then, saline aperients are the best and most efficacious; and where the disease does not admit of relief by surgical interference, the physician would best consult the interest of his patient, by urging him to reside at Cheltenham or Leamington, and by the aid of warm bathing and of drinking the waters regularly, to avail himself of the means which will most certainly mitigate his sufferings and prolong his life."

